# Lenvatinib combined with immune checkpoint inhibitors for unresectable, recurrent, or metastatic hepatocellular carcinoma: a real-world study

**DOI:** 10.1093/oncolo/oyag097

**Published:** 2026-03-20

**Authors:** Huifang Kong, Yan Chen, Hong Li, Di Wang, Xiujuan Chang, Zhen Zeng, Wei Zhang

**Affiliations:** Senior Department of Liver Disease Medicine, Chinese PLA General Hospital, Beijing, 100039, People’s Republic of China; Senior Department of Liver Disease Medicine, Chinese PLA General Hospital, Beijing, 100039, People’s Republic of China; Department of Infectious Diseases, Guizhou Provincial People’s Hospital, Guiyang, Guizhou Province 550499, People’s Republic of China; Senior Department of Liver Disease Medicine, Chinese PLA General Hospital, Beijing, 100039, People’s Republic of China; Senior Department of Liver Disease Medicine, Chinese PLA General Hospital, Beijing, 100039, People’s Republic of China; Senior Department of Liver Disease Medicine, Chinese PLA General Hospital, Beijing, 100039, People’s Republic of China; Senior Department of Liver Disease Medicine, Beijing Hospital of Integrated Traditional Chinese and Western Medicine, Beijing, 100039, People’s Republic of China

**Keywords:** hepatocellular carcinoma, lenvatinib, immune checkpoint inhibitors, real-world evidence

## Abstract

**Background:**

To investigate the outcomes of combined lenvatinib plus immune checkpoint inhibitors (ICIs) in patients with unresectable, recurrent, or metastatic hepatocellular carcinoma (HCC) in a real-world setting.

**Material and methods:**

This retrospective study included patients with unresectable, recurrent, or metastatic HCC who received lenvatinib combined with ICIs at the Fifth Medical Center of the Chinese PLA General Hospital between May 2018 and November 2022. The study outcomes were overall survival (OS), progression-free survival (PFS), and treatment response.

**Results:**

This study included 117 patients. The objective response rate (comprising both complete and partial responses) was 53.2%. Among those with first-line lenvatinib plus ICI (*n* = 109), the disease control rate (complete response, partial response, and stable disease) was 89.9%. In all patients, the median OS and PFS were 26.0 (95% CI, 22.0-30.4) and 15.3 (95% CI, 13.2-17.5) months, respectively. In first-line patients, the median OS and PFS were 27.6 (95% CI, 23.4-34.6) and 15.4 (95% CI, 13.4-18.2) months, respectively. Among the 117 patients, treatment was discontinued in 58 (50%) because of AE (*n* = 9, 16%), PD (*n* = 43, 74%), or an unknown reason (*n* = 6, 10%). Among the 117 patients, treatment was interrupted in 26 (22%), and the dose was adjusted in 24 (21%).

**Conclusion:**

This real-world study supports the possibility of using lenvatinib combined with ICI for the management of patients with unresectable, recurrent, or metastatic HCC, including first-line treatment. Confirmation through a formal clinical trial would provide firmer conclusions.

Implications for Practice This real-world evidence supports lenvatinib combined with immune checkpoint inhibitors as a possible effective and tolerable regimen for unresectable, recurrent, or metastatic hepatocellular carcinoma, including in the first-line setting. However, the findings need to be confirmed by formal clinical trials.

## Introduction

Hepatocellular carcinoma (HCC) is a highly lethal invasive carcinoma arising in the liver, accounting for the majority of primary liver cancers.^1^^,^^2^ There were 865 269 new cases of liver cancer in 2022 worldwide (6th most common cancer) and 757 948 related deaths (3^rd^ rank among cancer-related deaths).^3^ HCC is often unresectable or metastatic at presentation (10%-15% of cases).^4^ and prone to recurrence after curative treatment.^5^ The management of HCC is multidisciplinary and involves a combination of surgery, chemotherapy, radiotherapy, targeted therapy, and immunotherapy.^1^^,^^6^

Lenvatinib is a first-line targeted therapy approved for the management of unresectable HCC, providing comparable or better tumor response and survival outcomes than sorafenib, with a similar safety profile and potential utility in combination regimens.^7^^,^^8^ Immunotherapy represents a shift in the HCC management paradigm, particularly for advanced and recurrent disease, by improving survival rates and enabling long-term cancer control in subsets of patients, while generally minimizing adverse events (AEs) compared to traditional systemic therapies.^9^^,^^10^ Lenvatinib, combined with immune checkpoint inhibitors (ICIs), particularly with PD-1/PD-L1 inhibitors such as pembrolizumab, has emerged as a promising approach for advanced and unresectable HCC, offering improved antitumor activity, higher response rates, and an acceptable safety profile compared with either monotherapy.^11–15^

Still, it appears that the survival benefit of lenvatinib combined with ICIs versus lenvatinib alone is nuanced and context-dependent.^11–15^ Highly-selected patient populations in clinical trials for hepatocellular carcinoma (HCC) optimize internal validity by reducing confounders and enabling clearer assessment of treatment effects, but this comes at the cost of lowered generalizability to broader patient populations, particularly those with diverse comorbidities, advanced liver dysfunction, or poor performance status who are common in real-world clinical practice.^16^^,^^17^ Most available real-world evidence suffers from small sample sizes, the sequential use of lenvatinib after ICI instead of concomitant therapy,^18^ combined with other modalities such as hepatic artery infusion chemotherapy (HAIC),^19^ or the use of combined therapy in advanced lines.^18^

Therefore, the present real-world study aimed to investigate the outcomes of combined lenvatinib plus ICI in patients with unresectable, recurrent, or metastatic HCC. The results could help improve the management of HCC in a real-world setting.

## Material and methods

### Study design and patients

This retrospective study included patients with unresectable, recurrent, or metastatic HCC who received lenvatinib combined with ICI at the Fifth Medical Center of the Chinese PLA General Hospital between May 2018 and November 2022. This study was approved by the Ethics Committee of the Fifth Medical Center of the Chinese PLA General Hospital (approval KY-2024-8-120-1). Given its retrospective nature, the requirement for informed consent was waived.

The inclusion criteria were (1) age ≥18 years, any sex, (2) histologically or radiologically confirmed HCC, (3) radiological evaluation indicating unresectable, recurrent, or metastatic disease (AJCC stage IV or locally advanced unresectable), (4) received at least one cycle of a lenvatinib + immune checkpoint inhibitor (ICI) regimen, (5) availability of retrievable baseline imaging (computed tomography (CT) or magnetic resonance imaging (MRI)) for efficacy assessment, (6) complete baseline laboratory data, including complete blood count, liver and renal function, coagulation profile, and thyroid function, (7) Eastern Cooperative Oncology Group (ECOG) performance status of 0-3,^20^ and (8) follow-up of at least 3 months or documentation of death.

The exclusion criteria were (1) use of lenvatinib or ICI as monotherapy, without combination or sequential therapy based on lenvatinib plus ICI, (2) prior treatment with the same targeted tyrosine kinase inhibitor (TKI) (eg, sorafenib, regorafenib, apatinib, etc.) or second- or further-line ICI before crossover to lenvatinib plus ICI, (3) another primary malignancy requiring treatment within the past 5 years, (4) baseline Child-Pugh class C^21^ or severe hepatic impairment (ALT/AST >5× ULN, TBil >3× ULN), (5) severe cardiovascular events within the past 3 months (NYHA class III-IV heart failure, myocardial infarction, uncontrolled hypertension ≥180/110 mmHg), (6) pregnant or breastfeeding women, or (7) missing ≥20% of key medical record or follow-up data, making treatment response or toxicity assessment impossible.

During the study period, the combined regimen could include lenvatinib at 8-12 mg/d (8 mg/d for body weight <60 kg; 12 mg/d for ≥60 kg) and ICIs like PD-1/PD-L1 monoclonal antibodies, including toripalimab (fixed dose of 240 mg, intravenous infusion, every 3 weeks), camrelizumab (fixed dose of 200 mg, intravenous infusion, every 3 weeks), sintilimab (fixed dose of 200 mg, intravenous infusion, every 3 weeks), and atezolizumab (fixed dose of 1200 mg, intravenous infusion, every 3 weeks). Combination with chemotherapy, targeted therapy, or local treatments (TACE, ablation, or radiotherapy) was permitted as background therapy.

### Data collection and definitions

The data collected in this study included patient demographic characteristics (age and sex), clinical characteristics (HBV/HCV infection, α-fetoprotein [AFP] levels, Child-Pugh class, and ECOG performance status), tumor features (number of tumors, microvascular invasion [MVI], metastases, and Barcelona Clinic Liver Cancer [BCLC] stage), treatment information (use of local therapy, treatment lines, ICI received, dose adjustments, treatment discontinuation, and toxicity), and patient outcomes (treatment response and death).

For patients with multiple lines of treatment, only the first-line treatment was analyzed. The study outcomes were overall survival (OS), progression-free survival (PFS), and treatment response. OS was defined as the time from the initiation of lenvatinib treatment to death from any cause. PFS was defined as the time from the initiation of lenvatinib treatment to disease progression or death, whichever occurred first. The follow-up cutoff date was December 2024. Treatment response was assessed according to RECIST v1.1 criteria as complete response (CR), partial response (PR), stable disease (SD), and progressive disease (PD).^22^ The objective response rate (ORR) was the proportion of patients with CR or PR. The disease control rate (DCR) was the proportion of patients with CR, PR, or SD. The exact AEs were not documented because of the risk of information bias (ie, the patients consulted other hospitals or clinics, and the information did not reach the study hospital). Therefore, dose adjustments and treatment discontinuation were used as surrogates for safety.

### Statistical analysis

All statistical analyses were performed using Jamovi 2.6.44 (https://www.jamovi.org). Continuous variables with a normal distribution were presented as means ± standard deviation, and those without a normal distribution were presented as medians (range). Categorical variables were expressed as frequencies (composition ratios or percentages). Kaplan–Meier (KM) curves were plotted to evaluate patient survival after treatment.

## Results

### Characteristics of the patients

During the study period, 117 patients with unresectable, recurrent, or metastatic HCC were included. Among them, 89 (76%) were <65 years old, 102 (87%) were male, 109 (93%) had HBV infection, and 10 (8.5%) had HCV infection. Thirty-one (26%) patients had metastatic HCC, while 86 (74%) had advanced non-metastatic HCC. Among the 117 patients, 112 (96%) underwent local therapy (ie, transarterial chemoembolization [TACE], ablation, or radiotherapy), and 109 (93%) received lenvatinib and ICI as first-line treatment; only those 109 patients were included in the response analysis (Table 1). In the present study, 96% of patients received at least one locoregional therapy (TACE 38%, microwave ablation 7.1%, radiotherapy 14%, and argon‑helium cryoablation 63%), and many underwent multiple modalities (Table 2).

**Table 1.** Baseline characteristics of the patients.

Characteristic	*N* = 117
**Age**	
** <65**	89 (76%)
** ≥65**	28 (24%)
**Sex**	
** Female**	15 (13%)
** Male**	102 (87%)
**Hepatitis B virus infection**	109 (93%)
**Hepatitis C virus infection**	10 (8.5%)
**α-fetoprotein (ng/mL)**	
** <400**	67 (57%)
** ≥400**	50 (43%)
**Child-Pugh**	
** A**	88 (75%)
** B**	29 (25%)
**ECOG performance status**	
** 0**	27 (23%)
** 1**	83 (71%)
** 2**	6 (5.1%)
** 3**	1 (0.9%)
**No. of tumors**	
** 1**	11 (9.4%)
** 2**	2 (1.7%)
** ≥3**	104 (89%)
**Microvascular invasion**	
** No**	66 (56%)
** Only Portal vein branches**	24 (21%)
** Only Portal vein trunk**	7 (6.0%)
** Portal vein trunk and branches**	20 (17%)
**Metastasis sites**	
** Adrenal**	2 (1.7%)
** Bone**	3 (2.6%)
** Lung**	8 (6.8%)
** Lymph node**	18 (15%)
** No**	86 (74%)
**BCLC stage**	
** B**	41 (35%)
** C**	76 (65%)
**Local therapy^a^**	112 (96%)
**Number of treatment lines**	
** First**	109 (93%)
** Second**	8 (6.8%)
**Immune checkpoint inhibitors**	
** Atezolizumab**	24 (21%)
** Camrelizumab**	64 (55%)
** Sintilimab**	10 (8.5%)
** Toripalimab**	19 (16%)
**Immunotherapy type**	
** PD-1 inhibitor**	93 (79%)
** PD-L1 inhibitor**	24 (21%)

a Including transarterial chemoembolization, ablation, or radiotherapy.

Abbreviations: BCLC, Barcelona Clinic Liver Cancer; ECOG, Eastern Cooperative Oncology Group; PD-1, programmed death 1; PD-L1, programmed death 1 ligand.

**Table 2.** Local therapies received by the patients.

	First line (*n* = 109)	Second line (*n* = 8)	Overall (*n* = 117)
**Microwave ablation**	8 (7.7)	0	8 (7.1)
**TACE**	41 (39)	1 (13)	42 (38)
**Radiation therapy**	14 (13)	2 (25)	16 (14)
**Argon-helium cryoablation**	64 (62)	7 (88)	71 (63)

A given patient may have received more than one type of local therapy.

### Treatment response

**Figure 1.**
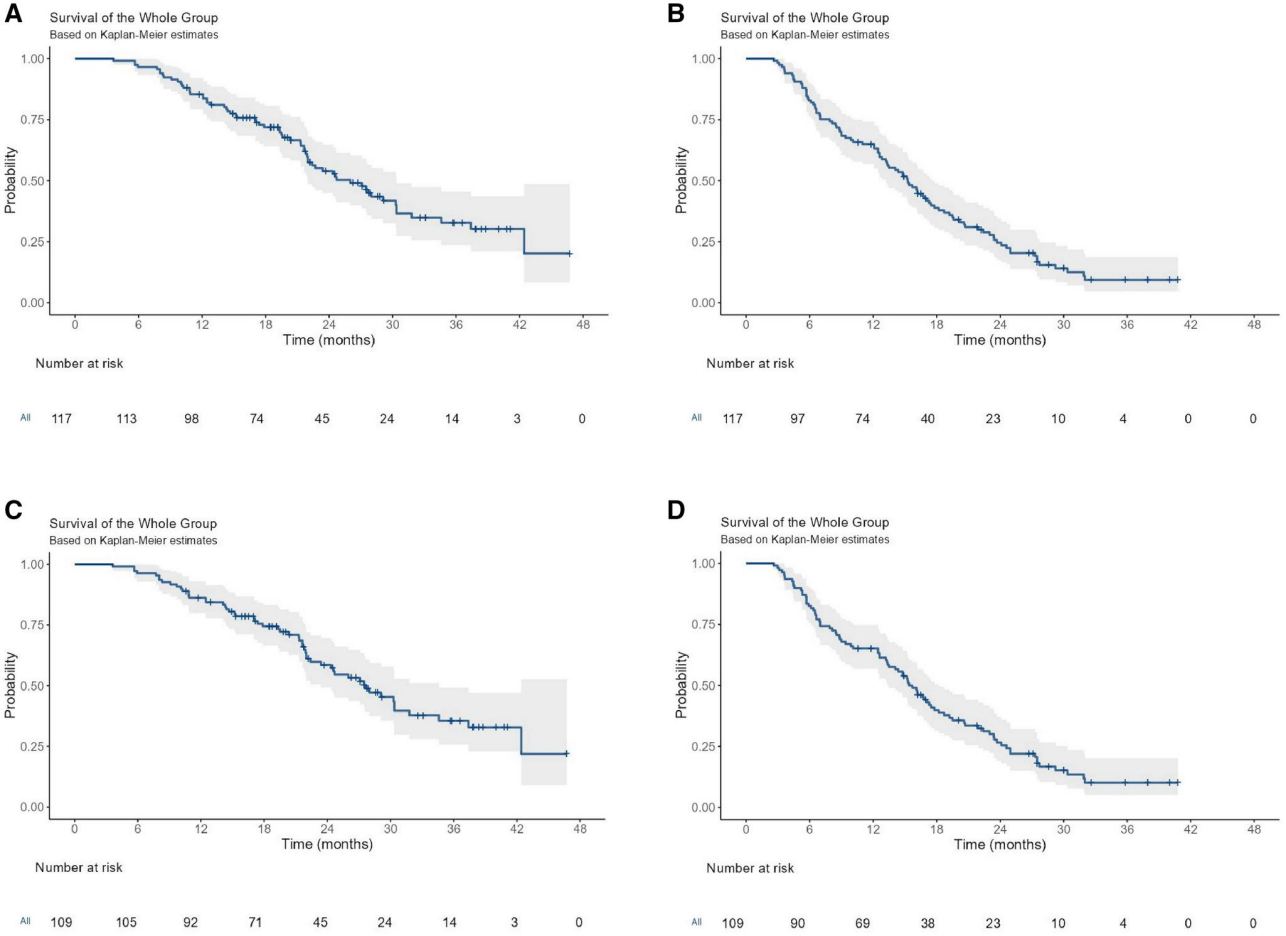
(A) Overall survival in all patients. (B) Progression-free survival in all patients. (C) Overall survival in first-line patients. (D) Progression-free survival in first-line patients.

As shown in Table 3, seven, 51, 40, and 11 displayed CR, PR, SD, and PD, respectively. Hence, the ORR was 53.2%, and the DCR was 89.9% (Table 2). In all patients, the 12- and 36-month OS rates were 85.4% and 32.8%, and the 6- and 12-month PFS rates were 82.9% and 64.9%. The median OS and PFS were 26.0 (95% CI, 22.0-30.4) and 15.3 (95% CI, 13.2-17.5) months, respectively. In first-line patients, the 12- and 36-month OS rates were 86.2% and 35.6%, and the 6- and 12-month PFS rates were 82.6% and 65.1%. Their median OS and PFS were 27.6 (95% CI, 23.4-34.6) and 15.4 (95% CI, 13.4-18.2) months, respectively (Figure 1).

**Table 3.** Treatment response to lenvatinib.

Response	*n* (*N* = 109)	%	95% CI
**Complete response**	7	6.4	3.1-12.7
**Partial response**	51	46.8	37.7-56.1
**Stable disease**	40	36.7	28.2-46.1
**Progressive disease**	11	10.1	5.7-17.2
**Overall response rate**	58	53.2	43.9-62.3
**Disease control rate**	98	89.9	82.8-94.3

### Dose adjustments

Among the 117 patients, treatment was discontinued in 58 (50%) because of AE (*n* = 9, 16%), PD (*n* = 43, 74%), or an unknown reason (*n* = 6, 10%). Among the 117 patients, treatment was interrupted in 26 (22%), and the dose was adjusted in 24 (21%) (Table 4).

**Table 4.** Lenvatinib dose adjustment or treatment discontinuation.

Characteristics	*N* = 117
**Drug discontinuation**	
** Yes**	58 (50%)
** No**	59 (50%)
**Reasons for drug discontinuation**	
** Adverse event**	9 (16%)
** Progressive disease**	43 (74%)
** Unknown**	6 (10%)
**Treatment interruption**	
** Yes**	26 (22%)
** No**	71 (61%)
** Unknown**	20 (17%)
**Dose adjustment**	
** Yes**	24 (21%)
** No**	69 (59%)
** Unknown**	24 (21%)

## Discussion

This real-world study supports the possibility of using lenvatinib combined with ICI for the management of patients with unresectable, recurrent, or metastatic HCC, including first-line treatment. Although the results are interesting, confirmation through a formal clinical trial would provide firmer conclusions.

The demographic profile from the study population (ie, primarily male, younger than 65, and overwhelmingly HBV-infected) mirrors findings from several large real-world studies of HCC. The predominance of HBV infection and male gender, as well as the age distribution, aligns closely with other published data from Asia-Pacific and global cohorts of patients with advanced or metastatic HCC.^23–26^ The global epidemiology considers that HCC is twice as common in males as in females.^2^ The present study found 26% with metastatic HCC and 74% with advanced non-metastatic disease. Real-world cohorts often focus on advanced unresectable HCC, with similar proportions of metastatic and locally advanced cases, though this varies depending on referral and selection criteria.^23^^,^^26^ Nevertheless, the study cohort reflects a highly selected, mostly HBV‑cirrhotic population with preserved liver function and many multifocal tumors. East Asia is an endemic region for HBV and HCV infections, driving the etiology of HCC.^27^ In many North American centers, the etiologic mix is more heterogeneous, with a higher proportion of HCV- and MASLD/ALD‑related HCC and a relatively lower proportion of HBV‑driven disease.^28^ These differences in etiology, stage at presentation, and liver function may influence natural history, transplant eligibility, and response/tolerance to systemic therapy and therefore should be acknowledged when extrapolating the study results to Western populations.^29^

The outcomes reported here for patients with unresectable, recurrent, or metastatic HCC, most of whom received lenvatinib and ICI as first-line treatment (ie, ORR of 53.2%, DCR of 89.9%, median OS of 27.6 months, and median PFS of 15.4 months), appear favorable and generally compare favorably with figures found in the literature, but with differences in patient populations (eg, second- or later-line therapy or after surgery). Indeed, studies and meta-analyses evaluating lenvatinib plus ICIs report an ORR for lenvatinib plus PD-1 inhibitors or combined therapies ranging typically from 24% to 53%, depending on cohort characteristics and combination regimen,^30^^,^^31^ aligning closely with the ORR of 53.2% observed in the present study. The DCR values reported in the literature for combination therapy range from 70% to 91%, which is similar to the results of the present study.^30^^,^^31^ A meta-analysis reported a median PFS of approximately 8.6 months.^31^ The median OS in large trials for combination therapy is often quoted around 22 months, supporting the OS rates observed here.^30^^,^^31^ However, in the present study, 96% of patients received at least one locoregional therapy (TACE 38%, microwave ablation 7.1%, radiotherapy 14%, and argon‑helium cryoablation 63%), and many underwent multiple modalities; these treatments were administered before and/or during systemic therapy according to real‑world practice patterns. Accordingly, the high objective response and disease control rates observed likely reflect the combined effect of locoregional therapy and lenvatinib plus ICI, rather than systemic therapy alone, and the exact relative contribution of each component cannot be determined in this retrospective, non‑randomized design. Therefore, the treatment benefits must be considered in the context of all treatments received by the patients, highlighting the need for individualized treatments centered on a common systemic strategy like lenvatinib plus ICI.

Patients in clinical trials are often selected, which may lead to different responses than those observed in real-world patients. Indeed, real-world studies of lenvatinib plus PD-1 inhibitors for unresectable HCC report ORRs between 19.6% and 29% and DCR of 73%-74%,^32^^,^^33^ lower than in the present study (ORR of 53.2% and DCR of 89.9%). In a real-world study of 296 patients with advanced HCC, those receiving lenvatinib plus a PD-1 inhibitor had significantly longer overall survival (median OS 20.1 months) and progression-free survival (median PFS not reported but improved) compared to lenvatinib alone (median OS 15.7 months); the 12, 24, 36-month survival rates were higher in the combination group (up to 79.1% at 12 months) than monotherapy groups, especially for patients receiving HAIC.^19^ The median PFS is commonly reported at 6.9 months in large Chinese cohorts, and as low as 3.7-5.2 months in multicenter North American and European studies. The 6- (82.9%) and 12-month (64.9%) PFS rates reflect better outcomes than those reported in other studies.^33^^,^^34^ Median OS is reported as 17.8 months in large Chinese real-world studies and about 12.8-14 months for North American cohorts. The 12- and 36-month OS rates (85.4% and 32.8%) are at the high end compared to these, where the 1-year OS is often 50%-70%, and the 3-year OS is around 30%-35%.^33^^,^^34^ Therefore, as highlighted above, despite all patients having unresectable, recurrent, or metastatic HCC, the favorable survival outcomes observed in the present study, compared with the literature, could result from the high use of local therapy (TACE, ablation, or radiotherapy) and the majority of patients being treated with lenvatinib plus ICI as first-line treatment. Younger age, better liver function, and lower tumor burden significantly improve response rates; however, real-world studies often include more heterogeneous patients with worse baseline health.^33^^,^^35^ Combination with local therapies (HAIC, TACE, or ablation), multidisciplinary management, and prompt initiation of second-line treatments can significantly improve outcomes above typical averages in broader real-world settings.^11^^,^^36^

Real-world experience indicates the combination of lenvatinib and ICI maintains a manageable safety profile, with expected AEs such as hypertension, proteinuria, and immune-related toxicities, and no significant new safety signals.^19^^,^^32^ In the present study, due to an information bias, the exact AEs could not be analyzed. Indeed, the study center is a reference center for HCC management, with many patients living at a certain distance from the hospital, and patients experiencing acute AEs are more likely to consult locally. Hence, the present study employed dose adjustments and treatment discontinuation to assess tolerability and safety, reporting rates comparable to those in the literature (31%-59%).^18^^,^^37^

In North America, a substantial proportion of patients with cirrhosis and HCC meeting standard criteria (eg, Milan: single lesion ≤5 cm or up to 3 nodules each ≤3 cm, no macrovascular invasion or extrahepatic spread) are evaluated early for liver transplantation, with established pathways for listing, downstaging, and bridging locoregional therapy.^38–40^ These programs often prioritize transplantation for patients within or successfully downstaged to Milan/UCSF‑type criteria, given excellent long‑term post‑transplant survival and low recurrence rates. In China and other parts of Asia, HBV‑related HCC is more prevalent, and patients frequently present with more advanced tumor burden or liver dysfunction, while organ scarcity, cultural factors, and heterogeneous center‑level criteria substantially limit access to liver transplantation.^41^ As a result, many patients who might be transplant candidates in North America instead receive systemic therapy and locoregional treatments as their main long‑term strategy, which partly explains why such a high proportion of the Chinese patients in the present study received lenvatinib plus ICI despite being potentially transplant‑eligible by North American criteria. These differences have two main implications for generalizability. (1) Transplant‑eligible patients managed non‑surgically: In North America, a significant subset of patients similar to the present patients (ie, non‑metastatic, Child‑Pugh A, and multifocal HCC) would undergo transplant evaluation and, if acceptable, be listed with bridging/downstaging therapy rather than receiving prolonged systemic treatment alone.^38–40^ Therefore, the survival and response outcomes observed in the present study represent a population in which systemic lenvatinib plus ICI is used, where transplant is not commonly available or prioritized, which may not fully reflect North American treatment sequences and decision‑making. (2) HBV‑dominant, Asian cohort: The predominance of HBV‑related cirrhosis and the local patterns of transplant scarcity and locoregional therapy use mean that the present real‑world cohort is most directly applicable to similar HBV‑endemic, organ‑limited settings. In regions with broader transplant access, different etiologic patterns, and alternative sequencing of curative vs systemic therapies, the magnitude and contextual meaning of the OS/PFS gains with lenvatinib plus ICI may differ.

This study has several strengths. It provides real‑world data on lenvatinib plus ICI in a relatively large single-center cohort of 117 patients with unresectable, recurrent, or metastatic HCC, including a clinically broad population with ECOG 0-3 and Child‑Pugh A-B, thereby enhancing generalizability beyond highly selected trial populations. Clear inclusion and exclusion criteria, together with standardized RECIST v1.1-based response assessment and conventional survival endpoints (OS, PFS, and landmark survival rates), support robust and comparable evaluation of treatment effectiveness. Restricting efficacy analyses to patients receiving the combination as first‑line therapy reduces heterogeneity related to prior systemic treatments, while detailed capture of locoregional modalities and systemic treatment modifications reflects contemporary multimodal practice and allows a nuanced appraisal of feasibility and tolerability using pragmatic surrogates such as dose adjustments, interruptions, and discontinuations.

The study had limitations besides the information bias related to AEs. The study was retrospective, limited to the data available in the charts or administrative databases. As it was a retrospective study, each patient was treated according to the patient’s disease and condition, the clinicians’ judgment, and applicable guidelines and policies, in opposition to a clinical trial with a predefined treatment strategy. Therefore, the patients received a variety of treatments around lenvatinib combined with ICI. Nevertheless, during the study period, in clinical practice, local treatment was usually carried out in conjunction with systemic treatment. Local treatments were done on an as-needed basis, and there was no clear distinction between pre-treatment, concurrent treatment, and post-treatment. The lack of standardized timing and sequencing between locoregional and systemic therapy is an important limitation and restricts causal attribution of efficacy to the systemic regimen itself; future prospective studies with predefined protocols and appropriate control arms (locoregional alone vs. locoregional plus systemic) are needed to clarify the incremental benefit of systemic therapy. Moreover, certain clinical factors potentially influencing HCC prognosis were not collected and analyzed. Meanwhile, the patients were from a single center, limiting the sample size and generalizability. Only a small number of patients received lenvatinib second-line therapy, which prevented further stratification analysis. Finally, no comparative group was included.

In conclusion, this real-world study supports the possibility of using lenvatinib combined with ICI for the management of patients with unresectable, recurrent, or metastatic HCC, including first-line treatment. Confirmation through a formal clinical trial would provide firmer conclusions.

## Data Availability

All data generated or analyzed during this study are included in this published article.
